# Evaluation of large language models on mental health: from knowledge test to illness diagnosis

**DOI:** 10.3389/fpsyt.2025.1646974

**Published:** 2025-08-06

**Authors:** Yijun Xu, Zhaoxi Fang, Weinan Lin, Yue Jiang, Wen Jin, Prasanalakshmi Balaji, Jiangda Wang, Ting Xia

**Affiliations:** ^1^ Department of Computer Science and Engineering, Shaoxing University, Shaoxing, China; ^2^ Institute of Artificial Intelligence, Shaoxing University, Shaoxing, China; ^3^ Department of Computer Science, College of Computer Science, King Khalid University, Abha, Saudi Arabia; ^4^ School of Life and Environmental Sciences, Shaoxing University, Shaoxing, China

**Keywords:** large language models, model evaluation, mental health, knowledge test, illness diagnosis

## Abstract

Large language models (LLMs) have opened up new possibilities in the field of mental health, offering applications in areas such as mental health assessment, psychological counseling, and education. This study systematically evaluates 15 state-of-the-art LLMs, including DeepSeekR1/V3 (March 24, 2025), GPT-4.1 (April 15, 2025), Llama4 (April 5, 2025), and QwQ (March 6, 2025, developed by Alibaba), on two key tasks: mental health knowledge testing and mental illness diagnosis in the Chinese context. We use publicly available datasets, including Dreaddit, SDCNL, and questions from the CAS Counsellor Qualification Exam. Results indicate that DeepSeek-R1, QwQ, and GPT-4.1 outperform other models in both knowledge accuracy and diagnostic performance. Our findings highlight the strengths and limitations of current LLMs in Chinese mental health scenarios and provide clear guidance for selecting and improving models in this sensitive domain.

## 1 Introduction

In recent years, large language models (LLMs) have made remarkable strides in the field of natural language processing, emerging as one of the most significant breakthroughs in artificial intelligence (AI) research. Since the introduction of the GPT (Generative Pretrained Transformer) model by OpenAI in 2018, LLMs have continued to evolve and expand (1). For instance, GPT-3, with its 175 billion parameters, and GPT-4, which incorporates multimodal processing capabilities, are able to understand and generate more complex, nuanced, and natural language texts. Concurrently, other research institutions and companies have also developed their own LLMs, such as DeepSeek-R1 (2), Google’s Gemma (3), and others. These models have demonstrated exceptional performance across a wide range of natural language processing tasks, including language understanding, text generation, and machine translation.

Given their ability to understand and produce human-like, fluent text, LLMs show significant promise in the mental health field, with potential applications in mental health assessment, psychological counseling, and education (4, 5). For example, LLMs can be used to develop emotionally supportive chatbots that offer instant emotional support and companionship to users (6–8). By analyzing users’ language or social media content, LLMs can detect emotional states and potential psychological issues, providing professionals with insights and even offering treatment suggestions and interventions (9–12). In the realm of mental health education, LLMs can generate tailored educational content to promote mental health awareness and knowledge (13, 14). These applications not only enhance the accessibility and efficiency of mental health services but also introduce innovative tools and methodologies for both research and practical applications in the field.

In addition to general-purpose LLMs, several domain-specific models have been developed for mental health tasks, include ZhiXin (15), MeChat (16), SoulChat (17), and MindChat (18). For instance, ZhiXin (15), a Chinese-language mental health model, is fine-tuned using psychiatric datasets and clinical guidelines. It outperforms state-of-the-art models in mental disorder diagnosis while maintaining safety, usability, and human-like responses. SoulChat Chen et al. (17), developed by the School of Future Technology at South China University of Technology and the Guangdong Provincial Key Laboratory of Digital Twin, is a large-scale psychological dialogue model. Through the fine-tuning of mixed long-text counseling instructions and multi-turn empathetic dialogue data, SoulChat significantly enhances the model’s ability to express empathy in psychological counseling scenarios.

While LLMs show great promise in mental health support, psychological assessment, and therapeutic assistance, the professional competence and potential risks associated with these models remain unclear. Therefore, it is essential to conduct thorough performance evaluations (19–22). Currently, there is a growing body of research focused on assessing the applicability of LLMs in the mental health field (23–27). A seminal study in this area was conducted by (23), which evaluated the zero-shot binary classification capabilities of GPT-3.5-Turbo in detecting stress, depression, and suicidality severity in social media text. Building on this work, the authors in (24) expanded the evaluation by examining GPT-3.5-Turbo’s performance in a broader range of affective computing tasks, including Big Five personality trait prediction, sentiment analysis, and suicide risk detection. In (25), the authors assessed the ability of LLMs to respond to a set of 18 psychological prompts to evaluate their potential applicability in mental health care. However, their study was limited to only two models: GPT-4 and ChatGPT.

Note that most of the studies mentioned above have used outdated models like GPT-3.5 and Llama 2 (24, 26). In contrast, recent months have seen the release of several highperformance LLMs, such as DeepSeek-R1, which in certain benchmark tests—such as mathematical reasoning and code generation—can match or even surpass the performance of GPT-4.0. Additionally, most of the studies focused on a single aspect when assessing the applicability of LLMs in mental health, such as evaluating the model’s conversational abilities or text generation quality. Some studies even employed manual evaluation methods (24, 25), which tested a limited number of models and tasks, and are inherently susceptible to subjective biases.

This study aims to evaluate the performance of state-of-the-art LLMs in Chinese mental health scenarios, focusing on two critical tasks: mental health knowledge assessment and mental illness diagnostic support. Our primary research questions are: (1) How do the latest LLMs compare in terms of their knowledge and diagnostic capabilities? (2) What factors influence their performance, beyond parameter size? We hypothesize that while larger models may generally perform better, architectural innovations and fine-tuning strategies could also play a significant role. This study addresses a gap in the literature by providing a comprehensive evaluation of the latest models, including DeepSeek-R1/V3 (March 24, 2025), GPT-4.1 (April 15, 2025), Llama4 (April 5, 2025), and QwQ (March 6, 2025, developed by Alibaba), which have not been extensively tested in this domain. Initially, we collaborate with professionals in the mental health field to curate representative test data, which includes public datasets from the web and professional literature datasets we use Chinese translations of public social media datasets obtained through web scraping. Automated tests were then conducted on the selected models using well-designed prompts. This detailed and holistic evaluation provides a more realistic and nuanced view of LLM performance in the complex, dynamic field of mental health applications, offering valuable insights for future model optimization and improvement.

## 2 Methods

### 2.1 Assessment tasks

This study systematically evaluates the performance of state-of-the-art LLMs, including DeepSeek-R1, GPT-4.1, and QwQ, across two critical tasks in the mental health domain: mental health knowledge assessment and mental illness diagnosis.

#### 2.1.1 Mental health knowledge test

The mental health knowledge test is designed to assess the LLM’s mastery and comprehension of mental health concepts. A comprehensive set of questions was gathered from the Chinese Academy of Sciences (CAS) Counsellor Qualification Exam (28), including both single-choice and multiple-choice questions. By analyzing the model’s responses, we can evaluate the depth and breadth of its knowledge base across various areas, such as personality psychology, social psychology, developmental psychology, and mental disorders. Furthermore, the model’s understanding of treatment methods—along with their applicable scenarios and principles—is evaluated. The results of this test will reveal whether the LLMs can provide accurate and reliable mental health knowledge, which is critical for its application in mental health education, awareness campaigns, and counseling services.

#### 2.1.2 Mental illness diagnostic test

This test evaluates the model’s ability to assist in diagnosing mental health conditions. We utilized publicly available social media datasets, including Dreaddit (29) and SDCNL (30), which feature posts related to mental health. The model analyzes the information in these posts and provides possible diagnostic results and recommendations for psychological disorders. The model’s performance is evaluated by comparing its diagnostic results to standard clinical diagnoses, calculating indicators such as accuracy and recall. This test is essential for evaluating the effectiveness of LLMs in clinical settings and offers valuable insights into their potential role in mental health diagnosis.

### 2.2 Datasets

To assess the application capabilities of LLMs in the field of mental health, we utilized a combination of publicly available high-quality datasets and custom-constructed dataset, which was collected using web crawlers. These strategies were implemented to ensure the reliability and validity of the evaluation outcomes.

#### 2.2.1 CAS counselor qualification exam dataset

The CAS Counselor Qualification Exam Dataset is derived from publicly available exam topics for the 2023–2024 period (28). It covers both theoretical and operational aspects of mental health. The theoretical topics include introduction to psychology, personality and social psychology, developmental psychology, mental health and mental disorders, and introduction to counseling. The operational skills section encompasses counseling theory, psychological assessment, basic counseling skills, and counseling methods. The dataset includes 744 single-choice and 200 multiple-choice questions.

#### 2.2.2 Dreaddit

Dreaddit (29) is a publicly available dataset designed for social media stress analysis. It consists of raw data from the Reddit community, spanning five forums (subreddits) focused on topics such as mental health, work stress, and related issues. The original dataset contains 190,000 posts, and all posts in this dataset had been pre-labeled by Amazon Mechanical Turk workers as part of the original dataset construction (29). We do not perform any additional annotation beyond using the provided labels.For this study, we selected a representative sample of 1,151 posts, including 64 labeled with “stress”, 503 with “anxiety”, and 584 with “PTSD”.

#### 2.2.3 SDCNL

SDCNL (30) is a dataset for the categorization of suicide and depression-related content. The dataset was compiled through web crawlers from two subreddits, r/SuicideWatch and r/Depression. The SDCNL dataset comprises 1517 labeled posts, including 787 annotated as “suicide” and 729 as “depression”, with balanced representation ensured during evaluation. This dataset is essential for evaluating the model’s ability to accurately classify content related to mental health crises.

### 2.3 Evaluated LLMs

To ensure a comprehensive evaluation of LLMs across diverse architectures and scales, this study incorporated a broad spectrum of models. The selected models included both state-of-the-art, large-scale models that excel in natural language processing tasks, as well as lightweight, resource-efficient models designed for deployment in constrained environments. In balancing cost and performance, we chose representative model families, including the DeepSeek series, OpenAI’s GPT series, Google’s models, and Meta’s Llama series, as shown in **Table 1**.

**Table 1:** List of LLMs.

No.	Model	Parameters	Release Date	Company	API Service
1	DeepSeek-R1	671B	January 20, 2025	DeepSeek	Silicon Flow
2	DeepSeek-v3(pro)	671B	March 24, 2025	DeepSeek	Silicon Flow
3	DeepSeek-R1-1.5B	1.5B	January 20, 2025	DeepSeek	Silicon Flow
4	DeepSeek-R1-32B	32B	January 20, 2025	DeepSeek	Silicon Flow
5	GPT-4o	200B	May 13, 2024	OpenAI	OpenAI
6	GPT 4.1	N/A	April 15, 2025	OpenAI	OpenAI
7	GLM-4 9B	9B	June 4, 2024	THUDM	Silicon Flow
8	GLM-4 32B	32B	April 14, 2025	THUDM	Silicon Flow
9	GLM-Z1 32B	32B	April 15, 2025	THUDM	Silicon Flow
10	Llama 3.3-70B	70B	January 29, 2024	Meta	Silicon Flow
11	Llama4-scout	17B	April 5, 2025	Meta	LlmAPI
12	Gemma-2-27b	27B	June 28, 2024	Google	Silicon Flow
13	Gemma-3-27b	27B	March 12, 2025	Google	LlmAPI
14	QwQ-32B	32B	March 6, 2025	Alibaba	Silicon Flow
15	Qwen2.5-72B	72B	September 18, 2024	Alibaba	Silicon Flow

### 2.4 Prompt engineering and evaluation setup

All models were tested via their respective APIs to ensure consistency and access to the latest versions. No temperature or system prompt settings were adjusted, as we aimed to evaluate the models’ default performance. For each prompt, only the first response generated by the model was considered. This approach was chosen to maintain a standardized evaluation process across all models. For each dataset, the mental health-related content was stored in.xls or.csv files, with each record represented as a string in a structured format (e.g., Reddit post content, exam questions, or metadata). These text strings were programmatically read into memory using Python-based tools. Each entry was then formatted into a task-specific prompt and sent to the LLMs via API. This process allowed us to automate the evaluation across multiple datasets and ensure consistency across model inputs.

## 3 Results

### 3.1 Mental health knowledge test

**Figure 1:**
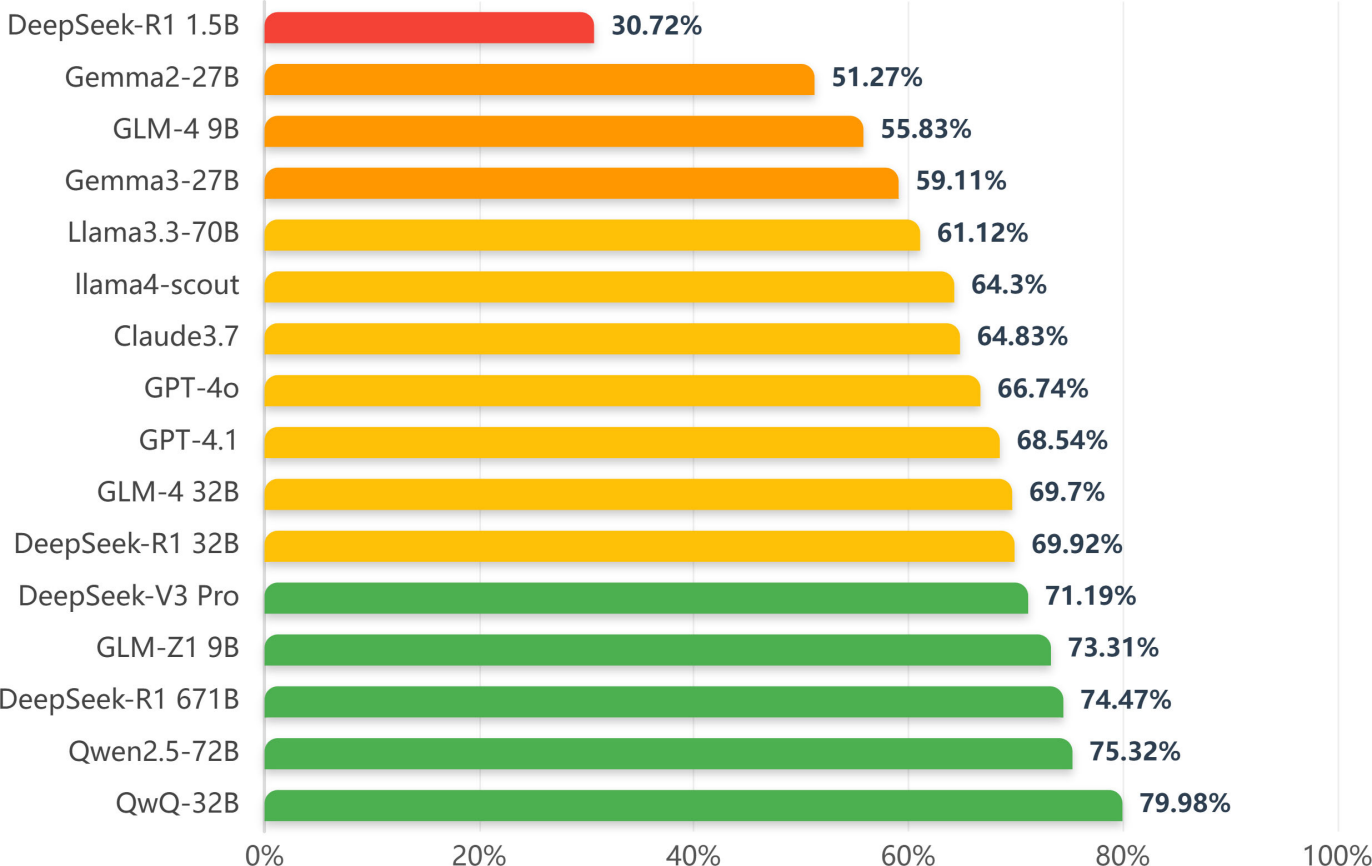
Overall accuracy of the knowledge test.

Firstly, we evaluate the performance of LLMs in the CAS Qualification Examination for Psychological Consultants. The dataset comprises 744 single-choice questions (SCQs) and 200 multiple-choice questions (MSQs). **Table 2** shows the test results of various models. For single-choice questions, DeepSeek-R1 671B and DeepSeek-V3 perform best, with accuracy rates of 86.83% and 84.68%. QwQ-32B, GLM-Z1, and GPT-4o also performed well, at 84.27%, 78.90%, and 72.72%. Generally, multiple-choice question accuracy is lower than single-choice. QwQ-32B has the highest multiple-choice accuracy at 64.0%, while DeepSeek-R1 and DeepSeek-V3 has only 28.5% and 21%. In terms of overall performance (see **Figure 1**), QwQ-32B and DeepSeek-R1 671B stand out with 79.98% and 74.47%.

**Table 2:** Results of mental health knowledge test.

Models	Single-Choice Questions	Multiple-Choice Questions
DeepSeek-R1 1.5B	35.08%	14.50%
DeepSeek-R1 32B	73.39%	57.00%
DeepSeek-R1 671B	86.83%	28.50%
DeepSeek-V3 Pro	84.68%	21.00%
Gemma2-27B	61.02%	15.00%
Gemma3-27B	64.52%	39.00%
GPT-4.1	73.52%	50.00%
GPT-4o	72.72%	44.50%
Llama4-scout	71.37%	38.00%
Llama3.3-70B	69.35%	30.50%
QwQ-32B	84.27%	64.00%
Qwen2.5-72B	81.45%	52.50%
GLM-4 32B	76.75%	43.50%
GLM-4 9B	64.38%	24.00%
GLM-Z1 9B	78.90%	52.50%

### 3.2 Mental illness diagnosis evaluation

In this task, we use Chinese translations of public social media datasets like Dreaddit (29) and SDCNL (30). Models analyze user-posted information to give possible mental disorder diagnoses. We conduct two tests: one using Dreaddit to judge depression, and another using SDCNL to assess suicide ideation. The test results are given in **Table 3**.

**Table 3:** Mental illness diagnosis test results.

Model	Suicidal Tendency Diagnosis	Depression Diagnosis
DeepSeek-R1 1.5B	51.91%	44.66%
DeepSeek-R1 32B	56.07%	56.39%
DeepSeek-R1 671B	67.15%	61.86%
DeepSeek-V3 Pro	64.45%	69.59%
Gemma2-27B	57.52%	72.02%
Gemma3-27B	59.76%	64.47%
GPT4.1	69.53%	61.51%
GPT-4o	49.93%	58.73%
Llama4-scout	61.41%	76.98%
Llama3.3-70B	57.98%	63.60%
QwQ-32B	58.44%	60.90%
Qwen2.5-72B	65.70%	59.51%
GLM-4 32B	62.14%	58.12%
GLM-4 9B	57.98%	56.65%
GLM-Z1 9B	61.54%	63.60%

In the suicide ideation diagnosis, model accuracy differs greatly. GPT-4.1 has the highest accuracy at 69.53%, much higher than average, showing strong risk identification. DeepSeek-R1 671B also performed well at 67.15%. Depression diagnosis performance varied more. Llama4-scout ranks first with 76.98% accuracy, followed by Gemma2- 27B (72.02%) and DeepSeek-V3 Pro (69.69%). Comparisons show that medium-scale models (e.g., Gemma2-27B) with certain optimization strategies outperformed some larger models. This suggests that model architecture and training strategy compatibility may be more effective than simply increasing parameter count. We also analyze failure cases in the diagnostic task and find that misclassifications often stemmed from symptom overlap (e.g., between anxiety and PTSD), ambiguous or metaphorical language, and lack of clinical context. The LLMs also tend to default to high-frequency categories like “anxiety” when uncertain. These issues highlight the limitations of using single-turn, text-only inputs for complex mental health assessments.

## 4 Discussion

The evaluation results revealed significant performance variations among models across tasks. In mental health knowledge tests, QwQ-32B achieved the highest accuracy of 79.98%, with 84.27% and 64.00% for single-and multiple-choice questions. In mental disorder diagnosis, GPT4.1 led in suicidal tendency diagnosis (69.53%), and llama4-scout in depression diagnosis (76.98%). Typically, model scale and parameter count positively correlate with knowledge and pattern learning ability, potentially leading to better knowledge-testing performance. For instance, DeepSeek-R1 1.5B’s smaller parameter count results in relatively low accuracy in both question types. However, larger models like DeepSeek-R1 671B don’t have the highest multiple-choice accuracy, indicating that model scale isn’t the only factor determining performance in all knowledge-based scenarios. Moreover, the low multiple-choice accuracy reveals that these LLMs still have significant limitations in handling complex mental health-related issues.

Beyond parameter size, architectural innovations, fine-tuning, and training datasets play crucial roles in determining model performance. For instance, medium-scale models like Gemma2-27B may outperform larger models due to their optimized architectures and training strategies. These factors enable models to better capture the nuances of mental health-related language and improve diagnostic accuracy.

One limitation of our current approach is the reliance on social media text (e.g., Reddit) for mental illness diagnosis. While informative, such data may not fully represent clinical presentation. Future work will incorporate electronic health records (EHR) and clinical notes to improve realism and relevance. Collaborations with certified psychologists and psychiatrists will also be pursued for expert validation.

It is also worth noting that using LLMs in psychiatry poses significant ethical risks. Hallucination (generation of false or misleading information) can lead to incorrect diagnoses or harmful suggestions. Additionally, LLMs may reflect biases from their training data, reinforcing stereotypes or minimizing serious symptoms. Privacy is another critical concern, especially when processing personal or clinical data. We emphasize that LLM-generated outputs must be reviewed by qualified mental health professionals and accompanied by strong data governance practices.

## 5 Conclusion

This study provides a comprehensive evaluation of 15 advanced LLMs in two key Chinese mental health tasks: knowledge assessment and illness diagnosis. Our results show that models like DeepSeek, QwQ-32B and GPT-4.1 outperform others in specific tasks, but significant limitations remain, particularly in handling complex, nuanced, or ambiguous cases. Model size generally correlates with performance, but is not the sole determinant. Inconsistent accuracy on multiple-choice questions and misclassifications in diagnosis highlight the need for further improvement. These findings underscore both the potential and current limitations of LLMs in mental health applications. Future work should incorporate clinical data, domainspecific fine-tuning, and expert validation to build more reliable and ethically robust systems for real-world use.

## Data Availability

The original contributions presented in the study are included in the article/supplementary material. Further inquiries can be directed to the corresponding author.
